# Carbon Sequestration in Turfgrass–Soil Systems

**DOI:** 10.3390/plants11192478

**Published:** 2022-09-22

**Authors:** Ruying Wang, Clint M. Mattox, Claire L. Phillips, Alec R. Kowalewski

**Affiliations:** 1 Department of Horticulture, Oregon State University, Corvallis, OR 97331, USA; 2 USDA-ARS, Northwest Sustainable Agroecosystems Research Unit, Pullman, WA 99164, USA

**Keywords:** greenhouse gas, soil organic carbon, biomass, photosynthesis, respiration, lawn, management, net ecosystem exchange, hidden carbon cost

## Abstract

Plants are key components of the terrestrial ecosystem carbon cycle. Atmospheric CO_2_ is assimilated through photosynthesis and stored in plant biomass and in the soil. The use of turfgrass is expanding due to the increasing human population and urbanization. In this review, we summarize recent carbon sequestration research in turfgrass and compare turfgrass systems to other plant systems. The soil organic carbon (SOC) stored in turfgrass systems is comparable to that in other natural and agricultural systems. Turfgrass systems are generally carbon-neutral or carbon sinks, with the exception of intensively managed areas, such as golf course greens and athletic fields. Turfgrass used in other areas, such as golf course fairways and roughs, parks, and home lawns, has the potential to contribute to carbon sequestration if proper management practices are implemented. High management inputs can increase the biomass productivity of turfgrass but do not guarantee higher SOC compared to low management inputs. Additionally, choosing the appropriate turfgrass species that are well adapted to the local climate and tolerant to stresses can maximize CO_2_ assimilation and biomass productivity, although other factors, such as soil respiration, can considerably affect SOC. Future research is needed to document the complete carbon footprint, as well as to identify best management practices and appropriate turfgrass species to enhance carbon sequestration in turfgrass systems.

## 1. Introduction

Carbon dioxide (CO_2_), methane (CH_4_), nitrous oxide (N_2_O), and fluorinated gases are greenhouse gases (GHGs) that contribute to global warming. The GHG with the highest concentration in the atmosphere is CO_2_, which contributed 81% of the total GHG emissions in 2018 [1]. In the ecosystem, plants are crucial players involved in carbon sequestration, which is the process of capture and storage of atmospheric CO_2_. While all living organisms release CO_2_ by respiration, atmospheric CO_2_ only enters the terrestrial ecosystems through photosynthesis of plants [2]. Plants assimilate CO_2_, store carbon in plant biomass, and contribute organic matter to soils. However, plants and soils also produce CO_2_ through respiration, and terrestrial ecosystems can be net sources of CO_2_ when they lose more stored carbon than CO_2_ taken in through photosynthesis on an annual basis.

A wide range of methods and terminology is used in the carbon research literature [3,4]. Measuring changes in soil organic carbon (SOC) over a period of time is a way to determine whether an ecosystem is a net sink or source, which is often expressed in the unit of Mg C m^−2^ yr^−1^ (conversion can be made using Table 1). Net ecosystem CO_2_ exchange (NEE) is another measure of whether a plant–soil system is a net sink or source of atmospheric CO_2_ at an annual time step. More importantly, whether a positive or negative NEE value indicates a sink of atmospheric CO_2_ needs to be specified. Over short time scales (<10 years), NEE provides a more sensitive approach for quantifying carbon sequestration than measuring changes in SOC. The fluxes of CO_2_ can be measured regularly with sealed gas chambers or with eddy covariance systems to estimate annual NEE. The units of SOC accumulation rate and NEE are in either weight of elemental carbon (C) or CO_2_ per area per year (Table 1).

Turfgrass covers an estimated 12.8 to 20 million ha of land in the United States [5], which will likely increase with human population and urban landscape growth. Turfgrasses are broadly used for sports (golf, football, soccer, baseball, tennis, etc.), residential and commercial areas (home lawns and commercial real estate), and public municipalities (parks, schools, and roadsides). In addition to their aesthetic value and functions, Morgan et al. [2] estimated that 5 Tg (1 Tg = 10^12^ g) of carbon was sequestered annually by turfgrass systems across the continental United States. Due to the higher soil carbon density relative to other land uses, residential lawns are potentially large pools for soil carbon [6,7,8]. However, maintaining high-quality turfgrass is reliant on repeated cultural practices, such as mowing, irrigation, and fertilization. Some intensively managed areas for sports, such as golf course tees and greens, as well as athletic fields, also require vertical cutting, aerification, sand topdressing, and pesticide applications. Fuel consumption and energy use for mowers and other machinery, irrigation pumps as well as production and transportation of fertilizers and pesticides for high-maintenance areas could offset the carbon sequestration benefits of turfgrass. Another concern associated with turfgrass management, like many agricultural systems, is the N_2_O emissions from irrigation and fertilization, which can contribute significantly to net GHG flux [9,10,11].

Due to the large range of turfgrass uses, species, age, and management practices, as well as the environmental settings in which turfgrass is grown, turfgrass can be a net source or a net sink of GHGs. The purpose of this literature review is to provide a general understanding of turfgrass systems, summarize current research on their climate impacts, and highlight potential ways to reduce their climate footprint. First, we describe the plant and soil components of turfgrass systems, as well as their carbon stocks and rate of carbon accumulation. Second, we compare carbon dynamics in turfgrass systems managed for different uses and compare turfgrass to other systems. Third, we summarize the key components that could affect carbon sequestration in turfgrass systems, including the age of turfgrass, grass species selection, turfgrass use, and management practices. Fourth, we provide an overview of methods used in studying turfgrass carbon dynamics for potential future research. Finally, we propose management practices that could potentially increase carbon gains and reduce carbon losses in turfgrass ecosystems.

## 2. Turfgrass Systems

**Figure 1:**
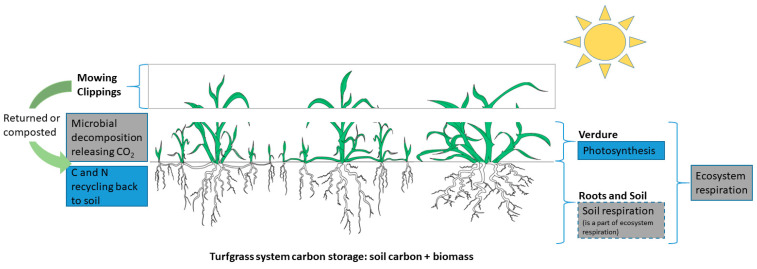
Biological components of the carbon cycle in a turfgrass–soil system. Blue boxes indicate carbon gains in the turfgrass system, and gray boxes indicate carbon losses in the turfgrass system or carbon emissions to the atmosphere. This figure describes common scenarios in which clippings are returned or composted to be added back to the soil. Some rare scenarios are not described in this figure, such as when clippings are burnt and the carbon captured in clippings is released into the atmosphere.

Turfgrasses are perennial plants that have long growing seasons and form a uniform ground cover when managed properly. In the turfgrass ecosystem, the uptake of atmospheric CO_2_ through photosynthesis occurs in the shoots under light, whereas respiration of the turfgrass (shoots and roots) and soil respiration contribute to the release of CO_2_ under light and dark conditions (Figure 1). Unlike forage grasses, other crops, and woody plants, turfgrasses are not bred or grown for high aboveground biomass yields, which would require increased mowing inputs. Therefore, turfgrasses are expected to store smaller amounts of carbon as aboveground plant biomass [12]. An extensive root system is an important trait for turfgrass to sustain adverse stress conditions [13]. However, when root turnover rate is taken into consideration, the carbon stored in the root biomass may not be a reliable carbon pool. High turnover rates of turfgrass roots indicate that roots are rapidly decomposed and turned over approximately every two years [12,14,15]. The carbon in turfgrass systems is therefore primarily stored in the soil as organic carbon. The SOC in turfgrass soils usually decreases with soil depth, and the most rapid accumulation usually occurs near the soil surface [16,17,18,19,20].

### 2.1. Soil Organic Carbon Stocks

In the literature, turfgrass lawns are generally reported to be carbon sinks, with the caveat that management practices can considerably affect carbon production and storage. Fine-textured soils with high clay content are better at stabilizing SOC and reducing the rate of decomposition [21]; however, soils with high clay content are prone to compaction and are therefore not suitable for turfgrass under traffic, such as sports turf and golf courses. For this reason, sports fields, as well as golf course greens and tees, are commonly constructed using sand and typically have less SOC than lawns grown on native soils [22,23]. However, research has shown that soil texture does not always have a significant influence on SOC stocks in residential lawns [16,17,24,25].

Wide ranges have been reported for turfgrass SOC stocks due to the wide range of environmental settings in which turfgrasses are grown. Selhorst and Lal [18] reported a mean SOC stock of 45.8 ± 3.5 Mg C ha^−1^ in various cities in the USA, ranging from 20.8 to 96.3 Mg C ha^−1^. Another commonly used unit in the literature for SOC stocks is kg m^−2^; for consistency with carbon sequestration rates reported in Mg C ha^−1^ yr^−1^, SOC stocks were converted to Mg C ha^−1^ by multiplying kg m^−2^ by 10 (1 kg m^−2^ = 10 Mg ha^−1^). In line with the study by Selhorst and Lal [18], studies on mature residential lawns have also reported a wide range of carbon stocks of 155 [26], 108.3 [24], 69.5 [20], 65.0 [27], 50.2 [17], 38.6 [16], and 19.7 Mg C ha^−1^ [28]. Pouyat et al. [6] compiled data from multiple cities and estimated mean SOC stocks of 71 and 144 Mg C ha^−1^ for parks and residential turfgrass, respectively. In New Zealand, Weissert et al. [29] reported a SOC stock of 48 Mg C ha^−1^ for urban parklands. When surveying 13 golf courses in southeastern suburbs of Melbourne, Australia, Livesley et al. [30] reported that SOC density varied from 49.8 to 147.5 Mg C ha^−1^ in rough and fairway soils. Other urban turfgrass soils (including park lawns, campus lawns, roadside turf, and athletic fields) were also reported as SOC stocks of 13–49 Mg C ha^−1^ to 15 cm depth [31] and 106–262 Mg C ha^−1^ to 1 m depth [32].

Despite the wide range in SOC stocks reported for turfgrass, studies have shown much more similar SOC stocks in residential lawns than in natural vegetation (such as forests, grasslands, and desert ecosystems depending on the climate) in cities with distinct climates [7,33]. For example, similar SOC stocks were reported between Baltimore, MD (110 Mg C ha^−1^), and Denver, CO (127 Mg C ha^−1^), residential turfgrass soils, likely due to the greater management efforts in the Denver region to offset the constraint of the dry climate [7]. In arid climates, turfgrass is often reported to have higher SOC stocks than native vegetation [7,33,34,35]. A study conducted on urban land use in Phoenix, AZ, also concluded that mesic landscaping with well-watered turfgrass was a net CO_2_ sink [36]. However, such studies highlight a tradeoff between water resources and the potential carbon sequestration benefits of turfgrass. While turfgrasses can accumulate large SOC stocks in arid climates, they require irrigation and other management practices. Using the CENTURY model to simulate turfgrass systems, Trammell et al. [37] demonstrated that management practices could be a potential driver for SOC accumulation. Research on turfgrass management practices is summarized and discussed separately in another section of this review.

### 2.2. Biomass and Net Primary Productivity

High SOC stocks in turfgrass systems are driven by high carbon inputs from plant biomass [38,39]. Newly seeded turf rapidly increased biomass carbon stocks; both aboveground and root biomass (1.8–3.4 and 1.0–2.2 Mg C ha^−1^, respectively) at three years after establishment were more than double the amount of biomass compared to one year after establishment [40]. Despite rapid growth rates, the amount of carbon stored in the turfgrass biomass was relatively low (2.4 [28] and 2.4–6.0 Mg C ha^−1^ [41]). Kong et al. [31] reported 0.5–2.1 Mg C ha^−1^ stored in turfgrass aboveground biomass as opposed to 12.6–48.9 Mg C ha^−1^ in the turfgrass soils.

Net primary productivity or production (NPP) is a measure of carbon inputs into an ecosystem. NPP can be calculated as the sum of the positive increments in the standing biomass, which requires periodic sampling. Falk (1980) proposed a calculation for NPP that uses turnover rates to estimate biomass production [15].

NPP = ∑clippings + stubble_max_ × *θ*_S_ + root_max_ × *θ*_R_, (1)

In this equation, NPP is the sum of the total clippings collected at each mowing, stubble production, and root production. Stubble or root production is calculated by multiplying maximum biomass (stubble_max_ or root_max_, respectively) by a turnover rate for stubble (*θ*_S_ or *θ*_R_, respectively). In that study, root and stubble turnover rates were measured, and an average NPP of 16.5 Mg ha^−1^ was reported in dry weight for lawns [15]. Qian et al. [42] also reported biomass allocations of 4.70, 3.37, 8.08, and 3.25 Mg ha^−1^ in biomass dry weight for clippings, verdure, thatch, and roots, respectively. Based on Equation (1) and turnover rates reported by Falk [14,15], Qian et al. [42] reported an NPP of 12.6 Mg ha^−1^ in biomass weight. However, these studies reported NPP in biomass dry weight; the amount of carbon in the biomass was not quantified and can vary depending on tissue type. The NPP rates in biomass weight can be converted to Mg C ha^−1^ yr^−1^ by multiplying by the appropriate carbon content (%) of each tissue type. For example, Golubiewski [34] reported that the carbon content of harvested clippings was 44.7% by weight. In another study, total standing biomass of a tall fescue [*Festuca arundinacea* Schreb. = *Schedonorus arundinaceus* (Schreb.) Dumort.] lawn averaged 6.04 Mg C ha^−1^ with slightly more carbon in roots than in stubble, and NPP averaged 4.50 Mg C ha^−1^ yr^−1^ [43]. Using a modeling approach, Milesi et al. [5] reported a wide range of NPP values from 0.22 to 10.6 Mg C ha^−1^ yr^−1^ associated with different management regimes.

**Figure 2:**
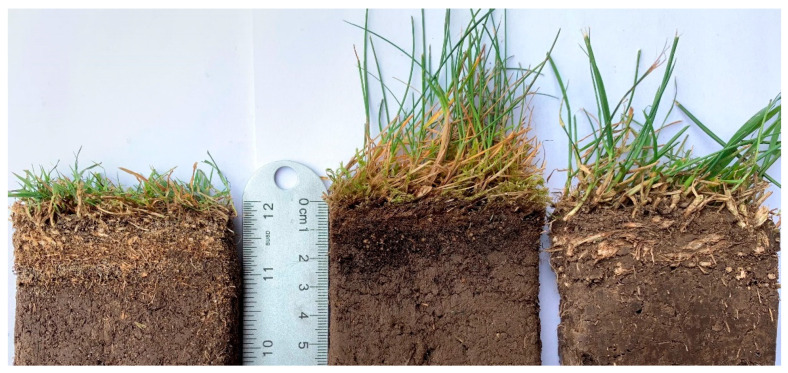
Turfgrass thatch development (approximately 2–3 cm as shown) in different turfgrass systems: creeping bentgrass (*Agrostis stolonifera*) maintained at a golf course fairway height (**left**), fine fescue (*Festuca* sp.) maintained as a lawn (**middle**), and tall fescue (*F. arundinacea*) maintained as a lawn (**right**).

It was unclear how much carbon was in thatch biomass in early turfgrass carbon research (for example, research by Falk in 1980 [15]), in which thatch might not be separated from other plant tissues when measuring standing biomass. This likely occurred because thatch was less commonly observed in older turfgrass cultivars (except for intensively managed areas, such as golf course putting greens). Benefiting from advances in turfgrass breeding, modern cultivars are denser and more aggressive in lateral growth than older cultivars [44]. Due to high plant density and lack of soil disturbance, turfgrass usually develops a distinct thatch or organic matter layer (Figure 2). Thatch in turfgrass has been defined as a layer of dead and living stems and roots that accumulates faster than decomposition between the green vegetation and the soil surface [45]. A study in 2020 reported that thatch built up rapidly after turfgrass establishment and contributed to carbon accumulation in turfgrass systems [46]. Turfgrass thatch layers have a higher carbon concentration (due to a higher lignin content) than verdure, roots, and underlying soils [47,48]. Therefore, thatch is a potential carbon pool in turfgrass systems [39,46,47].

Despite the fact that thatch layers are commonly observed in turfgrass systems, carbon studies vary as whether to include the thatch layer in determining SOC or total system carbon. The thatch layer has a comparable carbon content to that of soil [46,47]; therefore, this layer can also be a pool for carbon. A few studies have reported the carbon sequestration potential in thatch layers [39,42]. Thatch is commonly not included in soil carbon sequestration calculations [18,38,49,50,51]. Thatch has distinct physical and chemical properties different from verdure or roots. In Kentucky bluegrass (*Poa pratensis* L.) (rhizomatous), Qian et al. [42] separated thatch from verdure and roots and reported an annual thatch production (biomass of thatch × thatch turnover) of 4.362 Mg dry weight ha^−1^. Thatch has similar lignin content to that of roots and was therefore included as belowground biomass production [42]. Conversely, thatch and verdure have also been considered aboveground biomass [38,52]. Thatch can account for a substantial portion of the standing biomass, depending on grass species (more discussion is provided in a later section). However, thatch contributes to the softness of athletic fields; therefore, athletic fields require renovation and thatch removal to provide firm and smooth surfaces for the safety of players [53,54].

### 2.3. Ecosystem Respiration

Accumulation of carbon in turfgrass systems is controlled, in part, by carbon losses through respiration. The total plant, animal, and microbial respiratory loss of carbon from the ecosystem in the form of CO_2_ is defined as ecosystem respiration (R_eco_). Also referred to as total respiration, R_eco_ is composed of autotrophic respiration (R_a_) from plants and heterotrophic respiration (R_h_) from microbes and animals. Kong et al. [31] reported a lower R_eco_ (4.23 to 8.84 µmol m^−2^ s^−1^) in the dry season and higher rates (7.45 to 20.26 µmol m^−2^ s^−1^) in the wet season in Hong Kong. In a Singapore urban turfgrass system, Ng et al. [55] reported an R_eco_ rate of 7.9 µmol m^−2^ s^−1^, and R_a_ contributed a substantial portion. Simply converting respiration rates reported in µmol CO_2_ m^−2^ s^−1^ to an annual rate in Mg C ha^−1^ yr^−1^ is not appropriate if CO_2_ fluxes were only measured periodically or from a partial year because soil fluxes can vary considerably within a year. Song et al. [56] also reported a wide range of R_eco_ rates depending on mowing height and air temperature. Fertilization can also increase R_eco_ associated with turfgrass lawns [57]; whether elevated R_eco_ rates are the result of higher soil respiration or higher R_a_ from increased plant biomass in response to fertilization needs to be further investigated.

Ecosystem respiration can be equivalent to soil respiration in ecosystems without plants (such as bare soil) or in which plants (or plant parts) were removed when measuring respiration. However, many studies have not specified whether respiration from plants (R_a_) was included in soil respiration measurements. Studies quantifying respiration with sealed gas chambers have suggested that soil respiration contributes to CO_2_ emissions, also known as biogenic emissions, in turfgrass systems [29,40,55,58,59,60]. A few studies continuously surveyed CO_2_ fluxes for more than one year and calculated annual soil respiration rates of 10.5 [59], 9.2 [28], and 4.58 Mg C ha^−1^ yr^−1^ [61], which were converted to Mg C ha^−1^ yr^−1^ using Table 1 for ease of comparison to SOC accumulation rates. Using a modeling approach, R_h_ was estimated to be 0.31–1.21 Mg C ha^−1^ yr^−1^ with minimal management (mowing only as needed) and 1.38–9.22 Mg C ha^−1^ yr^−1^ under other management regimes on a nationwide scale in the USA [5]. Soil respiration from plant systems, including turfgrass, varies both spatially and temporally and can account for a substantial portion of urban carbon emissions [60]. Biogenic emissions measured from turfgrass soils were substantially higher than the fuel emissions from mowing [28,61].

Turfgrass thatch is a porous layer with stems and roots that also harbors macro- and micro-organisms [62,63] and is therefore expected to have a high respiration rate. Although the effects of turfgrass thatch on carbon sequestration are not fully understood, Raturi et al. [47] suggested significant differences in microbial biomass carbon between thatch and the soil underneath. Interestingly, thatch had higher microbial biomass carbon and lower carbon loss through maintenance respiration, suggesting that turfgrass thatch was acting as a temporary carbon sink, whereas the reduced microbial biomass and increased maintenance respiration associated with soils suggested that soils under thatch serve as sources of atmospheric CO_2_ [47]. Nevertheless, soil respiration is an important process for soil nutrient cycling and can serve as an indicator of microbial activities. Soil respiration from turfgrass systems was reported to be higher than that from bare soil [55,59,64], gravel mulch [65], and agricultural soils [35,64,66], indicating relatively higher microbial activities in turfgrass soils. Soil respiration rates measured in turfgrass systems are also comparable to other natural or managed ecosystems (Table 2) and were shown to be affected by soil temperature and moisture [29,59].

### 2.4. Hidden Carbon Cost and Net Greenhouse Gas Emissions

Although turfgrass systems continuously assimilate atmospheric CO_2_ through photosynthesis and accumulate SOC, there are concerns about turfgrass maintenance emissions, which can shift turfgrass systems from being carbon sinks to carbon sources [10,19,23,31]. Hidden carbon costs (HCCs) and net GHGs are expressed as CO_2_ equivalents (CO_2_-e) and are occasionally reported as C equivalents (C-e) in the literature, which are calculated by multiplying CO_2_-e values by 0.2727 (molecular weight of C/molecular weight of CO_2_). Some studies have estimated HCCs and GHGs in established turfgrass systems, accounting for fuel, irrigation, fertilization, and N_2_O emissions [23,72]. Zhang et al. [72] also included HCCs from production and transportation of pesticides, which accounted for the smallest portion among other factors. Two major types of turfgrass systems are lawns and golf courses, which can vary considerably in HCCs and net GHG emissions and are therefore discussed in detail in the following two sections.

Nitrous oxide (N_2_O) has a global warming potential (GWP) 298 times that of CO_2_. In turfgrass systems, N_2_O emissions related to fertilization and irrigation are a major component of net GHGs. Braun and Bremer [11] provided an in-depth review of N_2_O emissions in turfgrass systems and compared them to other crops and ecosystems. For the purpose of this review, we focus on the carbon cycle. Research on CH_4_ in turfgrass systems is limited, although a few assessments have indicated that CH_4_ fluxes are relatively small, except for during or immediately after rain or irrigation events [9,22]. Turfgrass systems are generally reported to be CH_4_-neutral or sinks [9,10,68,70,71].

#### 2.4.1. Lawns

Selhorst and Lal [18] demonstrated that lawns across the USA are potential sinks for atmospheric CO_2_; however, standard lawn management practices of mowing and fertilization contributed to HCCs of 0.190 and 0.064 Mg C-e ha^−1^ yr^−1^, respectively. Furthermore, Kong et al. [31] provided detailed HCCs of fuel use, electricity, irrigation, pesticides, and fertilizers associated with urban lawn maintenance, which contributed a total of 1.7 to 6.3 Mg C-e ha^−1^ yr^−1^ in carbon emissions. Such high HCCs can offset the carbon sink capacity of turfs in 5–24 years [31].

Ornamental lawns were reported to accumulate SOC at a rate of 1.40 Mg C ha^−1^ yr^−1^, which is greater than the GWP of N_2_O emissions in that system [23]. Therefore, the authors reported lawns sequestered CO_2_ at the rate of 0.29 Mg C-e ha^−1^ yr^−1^ under a low fertilization scenario (10 g N m^−2^ yr^−1^) after accounting for measured N_2_O emissions and estimated CO_2_ emissions generated by fuel combustion, fertilizer production, and irrigation [23]. However, under a high fertilizer scenario (75 g N m^−2^ yr^−1^), lawns were estimated to contribute to a carbon loss of 0.78 Mg C-e ha^−1^ yr^−1^. However, the 75 g N m^−2^ yr^−1^ of fertilizer applied to lawns is almost four times higher than the fertilization rate recommended by the local university extension office [73] and therefore not realistic. The reported net GHG also took N_2_O emissions into account, which were estimated to be 0.1 to 0.3 g N m^−2^ yr^−1^, depending on the fertilization rate and, when converted to GWP, resulted in +0.123 to +0.395 Mg C-e ha^−1^ yr^−1^ [23]. Similarly, Gu et al. [10] reported that carbon sequestration by turfgrass lawns was offset by N_2_O emissions and HCCs to maintain turfgrasses. In another case in Australia, when converting a well-established pasture to a turfgrass lawn, the turfgrass system was reported to produce net GHG emissions of 0.415 Mg CO_2_-e ha^−1^ (0.113 Mg C-e ha^−1^) in the first 80 days after conversion [9]. Therefore, understanding each plant–soil system is of great importance, and land conversion should be carefully considered.

#### 2.4.2. Golf Courses

Golf courses are unique turfgrass systems in which highly managed putting greens and tees account for only 5% of the average maintained turf acreage of 111.5 acres, whereas fairways and roughs account for 28.6% and 60% of golf course acreage, respectively [74]. Fairways and roughs are potential carbon sinks if such large-acreage turfgrass areas are managed with low inputs. For example, a golf course fairway turf in Manhattan, KS, was reported to have an average carbon sequestration rate of 1.01 Mg C ha^−1^ yr^−1^ [75]. In central Ohio, fairways and roughs were estimated to have sequestration rates of 3.55 and 2.64 Mg C ha^−1^ yr^−1^, respectively [19]. Large areas of fairways and roughs contributed to carbon sequestration, which offset the net emissions from greens and tees, with a net sequestration rate of the whole course of 1.47 and 0.44 Mg C-e ha^−1^ y^−1^ for a Parkland course and a Links course, respectively [76]. Additionally, naturalized roughs on golf courses are unmanaged areas covered by turfgrasses or a mixture of turfgrasses and other plants, which often do not require management inputs (no HCC). Despite the increasing popularity of such naturalized areas, owing to their environmental benefits [77,78], their carbon sequestration potential is largely unknown. We speculate that carbon stored in unmanaged roughs would be similar to that in the meadow-like lawns studied by Poeplau et al. [79] or unirrigated and mowed-as-needed roughs investigated by Qian et al. [38], which had less SOC than managed turfgrass areas. Studies in which the carbon budget for entire golf courses was calculated reported that golf courses were potential carbon sinks [76,80].

However, the emissions generated by maintenance can offset the carbon sequestration of turfgrass and trees on golf courses and should not be neglected. Selhorst and Lal [19] estimated large carbon losses (estimated 0.30 Mg C-e ha^−1^ yr^−1^) associated with maintenance practices, shifting golf courses from being carbon sinks to carbon sources within 30 years. The HCCs considered in their study included fertilizers, herbicides, insecticides, fungicides, irrigation, unleaded gasoline, and diesel fuel, with the highest HCC from diesel fuel combustion [19]. Bekken and Soldat [81] surveyed golf courses in the northern USA and estimated the total GHG emissions associated with maintenance to be 1.17 Mg C-e ha^−1^ yr^−1^, including onsite emissions (primarily fuel use), offsite emissions (primarily offsite electricity generation), and supply chain (upstream) emissions (primarily from the production and transport of machines, fertilizers, pesticides, etc.). Additionally, a few studies have reported detailed energy use and GHG emissions from management practices on greens, tees, fairways, and roughs [19,76,80,82]. Intensively managed turfs, such as golf course greens, consume energy and emit CO_2_ [76,80]. Carbon losses from turfgrass systems are often expected when aboveground tissues and underground organic matter are removed. Daily mowing with clippings removed when grasses are actively growing is a standard practice for golf course greens and tees [83]. In addition to removal of clippings by mowing, cultivation, including verticutting to remove grass tissues and hollow-tine aerification to physically remove plant materials and organic matter, is likely to reduce the carbon pool in turfgrass systems. Other practices, such as solid-tine aerification and topdressing, add sand to the soil profile without removing organic matter and plant material [62]. Such practices dilute the organic matter in the root zone profile to promote better growth of turfgrass and are therefore unlikely to reduce the productivity of turfgrasses. Research has been limited on the cultivation effects on the NPP and SOC of turfgrass, and the net carbon budget needs to be analyzed accounting for the HCCs of cultivation machine operations.

## 3. System Comparison

With increasing population and urbanization, vegetation and soil in the urban landscape are unable to balance the carbon emissions from human activities [84]. In urban landscapes, turfgrass helps to stabilize the soil, prevent wind and water erosion, and build up organic matter [85]. Urban turfgrass systems have received more carbon sequestration research attention compared to other turfgrass systems. Research on a nationwide scale in the USA has suggested that turfgrass systems in the urban landscape are potential carbon sinks [5,6,8,18,86], whereas many other studies have been conducted on smaller scales, such as cities, residential blocks, and individual lawns. Research by Qian and Follett [21] indicated the significance of turfgrass in carbon sequestration, which was comparable to USA lands in the Conservation Reserve Program. Gordon et al. [87] published a letter to the editor comparing turfgrass systems with other systems and concluded that turfgrasses are able to sequester CO_2_ at a rate similar to that of land used for agricultural and forestry practices, although carbon stored in the recalcitrant soil carbon pool is considered to be very limited due to the high turnover rate. In contrast to the large number of urban studies, very limited information is available on the carbon balance in agriculture systems where turfgrass sod and seeds are produced. Pahari et al. [88] reported that a warm-season turfgrass sod farm sequestered CO_2_ at a rate of 4.51–5.15 Mg C ha^−1^ yr^−1^. Research on the carbon footprint of turfgrass seed production is lacking.

Vegetative components of urban landscapes consist of trees, shrubs, herbaceous plants, and grasses. Comparing the impact of different urban landscape vegetation on carbon sequestration can be challenging for many reasons. Biomass can be directly measured in turfgrass systems, whereas it is often not feasible to harvest and measure above- and belowground biomass in systems with trees; instead, models are often used to estimate the biomass of trees. In addition, urban landscapes often receive carbon inputs on one landscape type from other onsite vegetation (such as tree leaves falling on a lawn) or from outside sources (such as compost additions in the urban landscape), making it difficult to derive the source of carbon in each system. Collecting data on two city blocks in Chicago, Jo and McPherson [12] concluded that larger carbon pools were stored in woody vegetation, such as trees and shrubs, compared to the intermediate pools of vegetation of turfgrass plants and no carbon storage in the herbaceous plants, whereas the majority of the carbon was stored in the soil (78.7% and 88.7% for the two blocks).

Soil organic carbon in the urban environment has also been explored (Table 2). Soil samples collected under tree canopies were shown to have higher SOC than samples from golf course fairways [30], whereas similar SOC values were observed between soils of turfgrass and trees in an urban landscape study [34]. Interestingly, lawns with trees were shown to have higher SOC at the 0–15 cm depth but similar SOC at soil depths of 15–30 cm and 30–50 cm when compared to lawns without trees [67]. These findings are contrary to the hypothesis that trees are expected to influence SOC at deeper soil depths because they have deeper root systems than turfgrasses. The authors also implied that turfgrass would be the main contributor to SOC at 0–15 cm [67]; therefore, one speculation is that tree canopies may have provided cooler and less stressful conditions than the full sun (possible heat stress environment) for turfgrass growth in the southern USA, where the study was conducted. A study in Auckland, New Zealand, compared ten urban forests dominated by trees with six urban parklands dominated by grasses; the authors concluded that the SOC was higher in the grass-dominated landscape (48 Mg ha^−1^) compared to the tree-dominated landscape (27 Mg ha^−1^) in the upper 10 cm [29]. Similarly, soil carbon density in the top 100 cm of residential soils was reported to be higher than in forest soils of similar types in a study conducted in Baltimore, MD [20].

Another landscape option is to grow non-turf herbaceous plants. A study in Germany sampled soils from 14 vegetable gardens and 13 lawns, revealing that vegetable patches contained a mean SOC stock of 164 Mg ha^−1^ and lawns contained 155 Mg ha^−1^ in the top 30 cm of soil compared to four samples from a local meadow, which contained 111 Mg ha^−1^ [26]. However, the ability to compare the SOC stock data between vegetable patches and lawns is complicated by the fact that lawn clippings and garden debris are often composted and later placed on vegetable patches. Vegetable gardens and mulch beds are common urban land cover options; such soils receive carbon additions, such as compost and wood mulch, and no differences were reported in SOC between these land covers and turfgrass [26,65,68].

Many research studies have compared turfgrass systems to adjacent ecosystems (Table 2). Higher SOC values in turfgrass systems compared to native grassland systems have been reported in numerous studies [7,26,34,35,64]. Moreover, lawns often have higher SOC values than agricultural soils [34,35,64,69], with the exception of one report showing similar SOC values between lawns and corn fields [66]. However, research conclusions in the literature are inconsistent when comparing forest with turfgrass ecosystems (Table 2). Forests are more complicated systems for carbon stocks, depending on the tree species (for example, deciduous broadleaf vs. evergreen needleleaf) and climate. Wildfire is another major concern with respect to carbon loss in forest ecosystems [89].

Comparing turfgrass sites to bare soil, Acuña E. et al. [50] reported that SOC increased over a 26-month period with nine turfgrasses in Chile, whereas the SOC in bare soil decreased (likely the labile SOC pool). This is consistent with other studies reporting higher SOC in lawns compared to bare soil [59,64]. Lawns also have higher soil respiration rates compared to bare soil [55,59,64]. Soil respiration, i.e., the process of releasing CO_2_ back to the atmosphere, represents a carbon loss from the plant–soil system. However, Bae and Ryu [59] reported that high soil respiration was correlated with high SOC stocks when comparing various systems: mixed forest, deciduous broadleaf forest, evergreen needleleaf forest, lawn, wetland, and bare land. One speculation is that high soil respiration is an indication of high microbial activities, which recycles nutrients from plant litter, subsequently adding carbon to the soil. Therefore, soil respiration alone cannot be the sole indicator of the net carbon balance of an ecosystem.

Higher soil respiration rates of lawns compared to agriculture lands and grasslands have been consistently reported in the literature [35,64,66]. There is no general agreement when comparing lawns with forests, likely due to spatial and temporal variations (Table 2). Wood-chip- or bark-mulched beds were shown to have similar high soil respiration rates relative to lawns [65,68]; such systems without plants do not have any carbon inputs from photosynthesis.

## 4. Age of Turfgrass

Numerous studies have reported higher SOC associated with older turfgrass systems, indicating the accumulation of SOC. Studies reporting SOC accumulation rates in turfgrass systems of varying ages are summarized in Table 3, which does not include studies utilizing model simulations (discussed in a separate section) or studies measuring SOC over time with repeated measurements. Carbon accumulation rates reported in studies with repeated measures over time were reported as 1.408 and 1.629 Mg C ha^−1^ yr^−1^ for Kentucky bluegrass and tall fescue, respectively [52]; 1.01 Mg C ha^−1^ yr^−1^ for zoysiagrass (*Zoysia japonica* Steud.) [75]; and 0.32, 0.74, and 0.78 Mg C ha^−1^ yr^−1^ for Kentucky bluegrass, fine fescue mixture (*Festuca* spp.), and creeping bentgrass (*Agrostis stolonifera* L.), respectively [38]. Soil total carbon accumulates over time; however, the ability of turfgrass systems to sequester and store carbon is not unlimited. Studies reported that carbon was linearly accumulated beneath turfgrasses over 33 years at a rate of 1.4 Mg C ha^−1^ yr^−1^ [23], 44 years at a rate 0.82 Mg C ha^−1^ yr^−1^ [20], 40 years at a rate of 0.69 Mg C ha^−1^ yr^−1^ [90], and 100 years at a rate of 0.30 Mg C ha^−1^ yr^−1^ [25]. As turfgrass ages, carbon is expected to reach an equilibrium in the system. Research has shown that initial SOC accumulation is greatest when turfgrasses are newly established; then, carbon sequestration rates decline as turfgrass systems age [10,21,42,49,91,92].

The rate of carbon accumulation and the time it takes for turfgrass systems to reach maximum carbon storage vary among turfgrasses depending on use (Table 3). Qian and Follett [21] analyzed the soil data of golf courses between the ages of 1.5 and 45 years and reported that rapid carbon sequestration occurred during the first 25 years after turfgrass establishment, at average rates of 0.9 to 1.0 Mg C ha^−1^ yr^−1^ to the 11.4-cm depth. In that study, soil carbon was reported to increase for approximately 45 years in putting greens and 31 years in fairways, as putting greens are established on sand with very low initial soil organic matter [21]. Other studies on putting green turf reported that SOC accumulation increased linearly in the top 25-cm soil at a rate of 0.69 Mg C ha^−1^ yr^−1^ for 40 years [90] and hyperbolically in the top 7.6-cm soil at a rate of 0.59 Mg C ha^−1^ yr^−1^ for 25 years [91]. Two studies on bermudagrass (*Cynodon* spp.) fairways also suggested a decreasing rate of carbon accumulation over time [49,94]. Soil carbon in the top 15 cm of fairways increased hyperbolically as accumulation rates declined from 6 Mg C ha^−1^ yr^−1^ to less than 0.5 Mg C ha^−1^ yr^−1^ in the first 20 years [94]. Gautam et al. [49] reported that soil carbon in the top 7.5 cm of fairways was accumulated at a rate of 0.22 Mg C ha^−1^ yr^−1^ and reached equilibrium after 46.4 years, whereas the 7.5–15 cm soil continued to sequester carbon for up to 62.5 years. Similarly, the time to attain equilibrium increased with an increase in soil depth; the time for the 0–2.5 cm soil of fairways and roughs to reach equilibrium was 14 and 12 years, respectively, whereas, the 10–15 cm soil depth was able to sequester carbon for up to 81 and 91 years, respectively [19].

Low rates of SOC were reported in residential lawns, with a linear accumulation of 0.29 Mg C ha^−1^ yr^−1^ at the 0–40 cm depth over the 100-yr chronosequence [25] and with a quadratic increase of 0.21 Mg C ha^−1^ yr^−1^ at the 0–10 cm depth for 53.6 years [93]. With 16 home lawn sites studied, Selhorst and Lal [18] revealed a wide range of SOC sequestered at the 0–15 cm depth, ranging from 0.9 to 5.4 Mg C ha^−1^ yr^−1^, depending on location. Land-use histories also alter the ability of residential lawns to sequester carbon. For instance, Raciti et al. [20] reported a rate of 0.82 Mg C ha^−1^ yr^−1^ accumulated in residential sites built on agricultural land but no correlation between age and SOC in lawns developed on forest land. One explanation they proposed is that residential lawns established on former forest land had higher initial soil carbon than those established on former agricultural land [20]. Campbell et al. [27] suggested that converting unmanaged Appalachian hardwood forests into managed residential lawns resulted in little change in the soil carbon of the upper 30 cm depth they sampled. Therefore, converting forests to residential lawns may not have any benefits with respect to SOC sequestration. Land-use history and land conversion are of considerable research interest; future meta-analysis is needed to elucidate the effects of land-use histories on carbon sequestration for decision making regarding land conversions.

Although numerous reports discussed above indicate that SOC accumulation rates decrease over time in turfgrass systems, there is no evidence of a notable decrease in turfgrass growth and carbon production. Shi et al. [94] summarized research results and implied that increased rates of soil organic matter degradation as turfgrass systems age are due to microbial activity. In support of this theory, microbial biomass and activity were found to be positively correlated with the accumulation of soil organic matter in aging turfgrass systems [95,96,97]. Although the accumulation rate seems to decrease, soil organic matter becomes more recalcitrant as turf increases in age [97].

In residential lawns, the accumulation of soil carbon over time is often reported in reference to the age of the home because house age is often an indicator of time since soil disturbance. In Salt Lake Valley, UT, SOC was reported to increase linearly with house age from 7 to 100 years [25]. In Manchester, NH, soil carbon stocks at 0–10, 10–20, 20–30, and 30–40 cm were positively correlated with house age [24]. In Colorado’s Front Range, residential sites >7 years had higher soil carbon concentrations in the surface soils (0–10 cm) than sites <7 years old, and homes >25 years in age had higher soil carbon concentrations in the subsurface soils (10–20 cm and 20–30 cm) than homes <25 years in age [34]. In Auburn lawns, soil carbon accumulated at low rates in the 0–15 cm depth (0.21 to 0.26 Mg C ha^−1^ yr^−1^) compared to other residential turfgrass studies, with no relationship with home age observed at the 15–30 and 30–50 cm depths [16,17]. In Montgomery County and Roanoke County, VA, Campbell et al. [27] reported a positive correlation between soil carbon concentration in the top 0–5 cm and time since residential parcel development (2–52 years). In an analysis of SOC data from 16 sites across the USA, Selhorst and Lal [18] indicated that home lawns did not have the benefit of sequestering carbon between 66 and 199 years with standard management practices, however, reduced inputs could further extend the time before emissions would cancel out sequestration.

In summary, turfgrass systems can accumulate SOC for 25 years or more (Table 3). Apart from being limited by the soil carbon capacity, turfgrass sites can deteriorate overtime due to pests, diseases, and weed invasion, which could contribute to a reduced sequestration rate. It is still unclear whether overseeding (with minimal soil disturbance) can affect carbon sequestration and extend the number of years for turfgrass systems to reach their carbon sequestration and storage capacity; therefore, future research is warranted.

## 5. Grass Species Selection

Many perennial grass species in the Poaceae family are used as turf and are adapted to a wide range of climates. Carbon stocks and sequestration rates can differ among turfgrass species. Acuña E. et al. [50] reported a range of SOC sequestration rates of 0.1–0.9 Mg C ha^−1^ yr^−1^ among turfgrass species tall fescue, strong creeping red fescue (*F. rubra* L. ssp. *rubra*), common bermuda (*C. dactylon* L.), hybrid bermuda (*C. dactylon* L. × *C. transvaalensis* Burtt Davy), Kentucky bluegrass, rough bluegrass (*P. trivialis* L.), and perennial ryegrass (*Lolium perenne* L.) in central Chile. By measuring seasonal physiological parameters, the authors found that in the summer, common bermuda (a C4 species) had high CO_2_ assimilation rates, low stomatal conductance, and high photosynthetic water use efficiency, which was calculated as the ratio between the simultaneously measured carbon gain in photosynthesis and water loss in transpiration. In the same study, tall fescue (a C3 species) maintained constant photosynthetic activity across all seasons. Both turfgrass species were shown to be promising species to increase carbon sequestration and to better use irrigation water in central Chile [50]. In another study, zoysiagrass was reported to have the highest mean levels of sequestered total carbon in biomass and soil when compared to other warm-season grasses (C4) for lawns, likely due to relatively higher shoot density [39]. In that study, zoysiagrass was reported to sequester carbon at a rate of 5.54 Mg C ha^−1^ yr^−1^ compared to 2.09 and 4.23 Mg C ha^−1^ yr^−1^ for hybrid bermuda and centipedegrass [*Erecholmoa ophroides* (Munroe) Hack.], respectively [39]. Turfgrass species with high shoot density are likely better at assimilating atmospheric CO_2_ (increased carbon inputs into the turfgrass–soil system). Therefore, high aboveground NPP is often correlated with high SOC [79]. On the other hand, high root biomass or high carbon allocation to root biomass likely contributes to greater SOC stocks [98,99]. This relationship of root biomass and SOC has not been clearly described in turfgrass. Hamido et al. [39] reported that the highest root biomass and root carbon were observed in zoysiagrass, followed by centipedegrass and hybrid bermuda, corresponding to their SOC sequestration. Using isotopes, Qian et al. [38] demonstrated that root biomass differences in hard fescue (*F. brevipila* Tracey) and sheep fescue (*F. ovina* L.) mixture, Kentucky bluegrass, and creeping bentgrass contributed significantly to SOC, although other factors could also affect the total SOC.

Whether cool-season (C3) and warm-season (C4) turfgrasses differ in carbon sequestration ability is still unclear. In a Mediterranean climate, common bermuda (C4) was shown to have higher photosynthetic capacity in the summer but was sensitive to mild or low temperatures; thus, there was no clear distinction between the carbon sequestration ability of C3 and C4 turfgrasses [50]. Another study indicated that common bermuda (C4) had lower SOC than tall fescue and Kentucky bluegrass (C3) in east Tennessee, likely because the higher temperature of the warm-season turfgrass growing season is also favorable for microbial decomposition of SOC [69]. A study of lawns with various turfgrass species in different climates suggested that higher SOC was associated with lower mean annual temperature [86]. Although temperature affects soil microbe activities and soil respiration, another possible factor is that cool-season grasses have a longer growing season compared to warm-season grasses, which become dormant during winter. Such speculation assumes cool- or warm- season turfgrasses are grown in the regions where they are adapted. Modeling the NEE of turfgrass on a nationwide scale, Milesi et al. [5] also implied that growing season length could affect the NPP of turfgrass.

The NPP and carbon allocation in turfgrass biomass can affect the carbon inputs in the turfgrass–soil system. Similar to Acuña E. et al. [50], Law et al. [100] reported that newly established (<3 years) tall fescue accumulated more labile soil carbon, total soil carbon, and soil organic matter than Kentucky bluegrass. In contrast, Law and Patton [52] evaluated tall fescue and Kentucky bluegrass cultivars with varying growth rates and concluded that in the short term, growth did not affect soil carbon accumulation but that slow-growing cultivars can have higher net carbon accumulation with less mowing requirements and fuel emissions. Qian et al. [38] quantified the soil carbon sequestration and SOC decomposition in C3 cool-season turfgrasses and reported higher net carbon sequestration rates for irrigated fine fescue rough (0.74 Mg C ha^−1^ yr^−1^) and creeping bentgrass fairway (0.78 Mg C ha^−1^ yr^−1^) than for Kentucky bluegrass short rough (0.32 Mg C ha^−1^ yr^−1^). Fine fescues were also shown to have great potential for soil carbon accumulation in the surface 20 cm profile relative to other C3 cool-season turfgrasses, which were ranked in the following order: red fescues (*F. rubra* spp.) > sheep fescue > creeping bentgrass, tall fescue, Kentucky bluegrass > perennial ryegrass [46]. Interestingly, such variation among turfgrass species and subspecies was related to thatch thickness [46]. In another study, carbon stored in the thatch layer varied from 0.05 to 0.1 Mg C·ha^−1^ yr^−1^ in the order of zoysiagrass < hybrid bermuda < centipedegrass lawns [39]. Zoysiagrass, hybrid bermuda, and centipedegrass are warm-season grasses that propagate by stolons and/or rhizomes.

Fast-growing and dense turfgrasses, as well as rigorous lateral growth type turfgrass species, often favor thatch development. Stolons are aboveground stems, whereas rhizomes are underground stems, both allowing turfgrass to spread horizontally. More importantly, stolons and rhizomes are major storage regions for carbohydrate reserves [101]. Creeping bentgrass (stoloniferous) and zoysiagrass (rhizomatous and stoloniferous) thatch was reported to have high carbon contents of 77.7 and 73.4 g kg^−1^, respectively, and the authors also suggested that thatch can be a temporary carbon sink [47]. The thatch biomass of Kentucky bluegrass, creeping bentgrass, and fine fescue (hard fescue and sheep fescue mixture) was greater than that of verdure or root biomass [38,42]. Additionally, Evers et al. [46] showed that carbon accumulation in the thatch/mat layers was higher than that in the 0–20 cm soil depth. Given that thatch has been shown to have high carbon content [48], whether turfgrass species with thatch-forming tendency have greater potential for carbon sequestration needs to be further investigated.

Research on the adaptation of turfgrass species on a nationwide or global scale is critically important but very limited. High CO_2_ assimilation rates and long growing seasons can be equally important when choosing turfgrass species. Turfgrass species that are adapted to local climates, as well as those that are tolerant to environmental (cold, heat, drought, etc.) and biotic (diseases, insects, etc.) stresses are able to maintain turf color and cover to assimilate atmospheric CO_2_ without going into dormancy under adverse conditions. The growth rate of turfgrass species is not a reliable indicator of carbon sequestration rate. Other factors, such as biomass production and allocation of carbon to shoots, roots, and thatch, also need to be considered. Enhancing carbon sequestration through grass species selection and adaptation is an important direction for future research.

## 6. Turf Use and Management Intensity

High management inputs often ensure healthy and dense turf, producing greater amounts of above- and belowground biomass, which increases primary productivity. Using models, a number of studies have predicted that increasing resource inputs (such as fertilization and irrigation) would increase carbon sequestration [5,10,42]. However, operations and maintenance contribute a significant portion of carbon emissions in the turfgrass carbon budget.

Home lawns vary considerably in terms of management practices and intensity. Despite the limited scale of research comparing two lawn sites, early research showed that more intensive management led to greater aboveground production but similar NPP [15]. Although changes in NPP were insignificant, Lilly et al. [43] demonstrated that maintenance practices had substantial effects on how carbon was allocated in the production of root, stubble, and clipping biomass. Additionally, Golubiewski [34] reported that high management increased the aboveground NPP and biomass. High maintenance ensures the density and quality of turfgrass, resulting in increased biomass. Using a modeling approach, Zirkle et al. [8] was able to analyze soil data on a large scale and concluded that low management with minimal input (mowing only) resulted in the lowest net SOC sequestration rate (accounting for HCC) of 0.254 to 1.142 Mg C ha^−1^ yr^−1^, whereas do-it-yourself management by homeowners and high management based on best management practices resulted in sequestration rates of 0.806 to 1.830 Mg C ha^−1^ yr^−1^ and 0.517 to 2.043 Mg C ha^−1^ yr^−1^, respectively. In another study, Gu et al. [10] showed that greater management intensity could contribute to higher SOC and higher net GHG emissions. Reducing management practice intensity could effectively reduce net GHGs and N_2_O emissions; however, lawns without irrigation and fertilization were gradually depleting the SOC pool [10].

In other cases, management practices have very limited effects on soil carbon [16,75]. Intensively managed turfs, such as golf course greens, consume energy and emit CO_2_ [76,80], whereas fairways and roughs require less input. Braun and Bremer [75] reported that a higher-input management (urea fertilization and medium irrigation regime) was shown to have higher HCCs and did not increase net carbon sequestration compared with a low management input (no N fertilization and low irrigation regime). High management intensity does not always guarantee carbon gains in turfgrass systems but contributes to significant HCCs; therefore, the effects of each management practice on carbon sequestration need to be evaluated.

## 7. Management Practices

Proper management practices are crucial for minimizing biotic and abiotic stresses in turfgrass. When turfgrass is under stress, respiration exceeds photosynthesis, resulting in CO_2_ release into the atmosphere. Irrigation, fertilization, and mowing practices can positively or negatively affect the ability of turfgrass systems to assimilate and store carbon. Many studies have evaluated the individual effects of irrigation, fertilization, and mowing or a combination of these cultural management practices.

Mowing is considered the most energy-consuming practice in turfgrass management [82]. Irrigation and fertilization are primary cultural practices that can promote the production of shoot and root biomass, as well as NPP, but also increase soil respiration [5]. Another concern is that irrigation and fertilization could lead to the emission of GHGs. Gu et al. [10] raised concerns about N_2_O emissions with irrigation and fertilization practices. Research by Livesley et al. [68] demonstrated that N_2_O emissions increased sharply and peaked following a fertilizer application and rainfall event. Braun and Bremer [11] provided a review of N_2_O research in turfgrass systems and reported a wide range of N_2_O emission factors (0.17% to 5.1%) of applied N fertilizer with an average of 1.9%. There is a need for research-based information to utilize management practices that increase carbon gains and reduce carbon costs.

### 7.1. Irrigation

Research showed that low soil water content (<0.15 m^3^ m^−3^) can limit the ability of turfgrass to assimilate atmospheric CO_2_ in response to high light intensity, whereas under adequate water soil conditions (>0.15 m^3^ m^−3^), the NEE of turfgrass increased as light intensity increased [88]. Under warm conditions, irrigation can also promote microbial activities, which consequently decompose soil organic matter. Therefore, irrigation was reported to increase both SOC input and decomposition [38].

Carbon balance affected by irrigation can vary considerably, depending on the climate and precipitation. The requirement for irrigation can be minimal in temperate regions where turfgrass is well adapted, whereas irrigation plays a vital role in arid and semiarid regions and can represent a major source of carbon consumption in turfgrass systems. The energy required for irrigation was estimated to be about 193 g CO_2_ m^−2^ yr^−1^ (0.526 Mg C-e ha^−1^ yr^−1^), which is higher than the estimated CO_2_ emissions from fuel consumption (122 g CO_2_ m^−2^ yr^−1^ converted to 0.333 Mg C-e ha^−1^ yr^−1^) for maintenance because this study was conducted in Irvine, CA, a moderately dry climate where annual precipitation is approximately 350 mm yr^−1^ [23]. In Phoenix, AZ, mesic landscaping with irrigated turfgrass was reported to be a carbon sink primarily controlled by plant photosynthetic activity, whereas other landscapes were unable to offset emissions from anthropogenic processes [36]. Research conducted in College Park, MD, a temperate climate with annual precipitation of 1065 mm yr^−1^, indicated that irrigation did not affect NPP but increased root biomass compared to no irrigation [43]. Qian et al. [38] demonstrated that carbon sequestration rates on a golf course in Nebraska City, NE, were 0.74 and 0.52 Mg C ha^−1^ yr^−1^ for irrigated and unirrigated (twice a week at 70% ET) fine fescue mixture, respectively; however, this is not a direct comparison, as irrigated and unirrigated fine fescue mixtures were maintained at different mowing heights (5.1 and 7.6 cm, respectively). Irrigation was reported to increase both aboveground NPP and SOC; therefore, a modeling approach by Zhang et al. [102] predicted a 50% reduction in the annual net production when irrigation was decreased from 100% to 60% potential evapotranspiration in the Colorado Front Range, a semiarid region.

### 7.2. Nitrogen Fertilization

Nitrogen (N) is the most important nutrient for turfgrass establishment and growth [103]. In addition to promoting above- and belowground biomass, N also affects stress tolerance to temperature and pests [103]. Without N fertilization, turfgrass struggles to maintain its overall quality and vigor. In carbon research, N was shown to promote carbon sequestration compared to no N [51]. However, N applications only contributed to the SOC increase in the soil surface at the 0–2.5 cm depth [51]. Similarly, increasing fertilization frequency was correlated with higher soil carbon content at the 0–5 cm depth [27]. Nitrogen primarily promotes aboveground biomass; hence, deposits of old leaves increase SOC at shallow soil depths.

On the contrary, increasing N rates may not be beneficial and can sometimes negatively affect carbon sequestration in turfgrass systems. Measuring soil respiration rates with an opaque closed gas chamber suggested that CO_2_ emissions significantly increased from 292 to 394 kg ha^−1^ d^−1^ as the N rate increased from 24 to 196 kg ha^−1^ in 8-yr-old ‘Tifway’ hybrid bermuda plots, and fertilization in association with higher soil temperatures and moisture contents resulted in larger fluxes of CO_2_ [58]. The authors speculated that N fertilization stimulated microbial and root activities, resulting in an increased CO_2_ flux from the soil [58]. Similarly, Brandani et al. [104] reported generally higher soil CO_2_ emissions as the N rate increased in tall fescue and hybrid bermuda research plots. While N is essential for newly established turfgrass, N rates can be reduced in mature turfgrass and still achieve similar carbon sequestration in the soil [42,72]. Reducing N fertilization also reduced N_2_O emissions [10,23,57], whereas fertilization did not affect soil CH_4_ exchange [68,104]. In summary, reducing fertilization can be an effective means of mitigating GHGs from turfgrass–soil systems [10,23].

Fertilization can affect carbon allocation in turfgrass systems, which also depends on the grass species. One study showed that fertilization did not influence the SOC concentration in a mixture of strong creeping red fescue and Kentucky bluegrass but increased the thickness of the thatch layer [105]. Likely because both species are aggressive rhizomatous type turfgrasses, carbohydrates are allocated in rhizomes for storage, resulting in thatch buildup rather than increasing SOC. Grass clippings decompose quickly, which can contribute to the SOC in the soil surface [106], whereas thatch is more resistant to decay than clippings or senescent leaves [48]. In tall fescue lawns, increasing N fertilization increased clippings production but did not affect the NPP when clippings were returned [43]. An increase in clipping biomass could lead to a significant carbon loss from the turfgrass system if clippings are removed. Clipping management is further discussed below. A higher-input management regime of irrigation and N fertilization did not increase carbon sequestration compared with a low management input regime, suggesting the potential of utilizing minimal maintenance practices to save energy [75]. Collectively, research has shown that N fertilization in turfgrass systems has limited benefits for carbon sequestration and GHG mitigation, especially with mature stands.

### 7.3. Mowing

Mowing can affect the biomass production of turfgrass, as well as soil respiration, by altering soil moisture and temperature. Mowing practices have received a considerable amount of research attention. The effects of mowing height, mowing frequency, and clipping management on carbon balance in turfgrass systems have been evaluated. Few studies have shown that mowing has a significant impact on carbon balance in turf [12,107].

Turfgrass managed under higher mowing height has greater shoot biomass and therefore greater capacity for carbon fixation through photosynthesis [56]. In addition to an increased photosynthetic rate, Kentucky bluegrass mowed at 7.6 cm generally had a higher R_eco_ rate and canopy photosynthesis to R_eco_ ratio compared with Kentucky bluegrass mowed at 3.8 cm [56]. R_eco_ includes respiration from shoots, roots, and soil microorganisms. Although a higher mowing height has greater potential to assimilate CO_2_ from the atmosphere, cool-season turfgrass can still act as a carbon emitter during warm months when the total respiration rate of shoots, roots, and soil exceeds canopy photosynthesis [56]. In another study, mowing height (5 or 10 cm) did not affect the NPP (sum of clippings, stubble, and root production) of tall fescue lawns [43].

Reducing mowing frequency reduces HCC from fuel consumption and can also affect respiration and aboveground NPP in turfgrass systems. Allaire et al. [107] reported that mowing frequency mostly influenced respiration (biogenic CO_2_ emission) as compared to N fertilization, and a frequently mowed turfgrass system produced CO_2_ emissions four times higher than an infrequently mowed turfgrass system. Interestingly, soil CO_2_ fluxes were unaffected by mowing frequency in another study, and fuel emissions from mowing were minimal compared to those from soil respiration [61]. Frequent mowing increased aboveground NPP and SOC compared to meadow-like lawns that were mowed once per season in some sites but not all six sites [79]. The authors also found that root biomass was not affected by mowing, suggesting that mowing could increase SOC by promoting aboveground NPP, which is a significant carbon input to turfgrass systems if clippings are left on the lawn [79]. To reduce the gasoline emissions associated with mowing, choosing an appropriate type of mower needs to be considered. Recently, battery-, electricity-powered mowers and manual reel mowers with much lower energy consumption have become popular alternatives to gasoline mowers [108,109,110].

Both returning and removing clippings are common mowing practices in turfgrass management. Grass clipping management affects the recycling of C and N and is therefore a crucial part of the carbon balance in turfgrass systems. Research has shown that a substantial amount of carbon fixation in turfgrass is allocated in producing aboveground biomass; therefore, clipping management can be a critical driver of the carbon balance in turfgrass systems [28,42,52]. Returning clippings was demonstrated to reduce net GHGs by 12% [10]. Grass clippings are a source of N; therefore, returning clippings could have a similar effect as adding N fertilizer. Qian et al. [42] reported that returning clippings increased soil carbon sequestration, and such an effect was more pronounced under a low fertilization regime. Returning clippings contributed to substantial increases in turfgrass productivity and small increases (0.2%) in SOC [111]. Additionally, increases in carbon content and stock due to returning clippings only occurred in the top 5 cm [105] and top 15 cm [17] soil layer but not in the deeper soil profile. Turfgrass clippings decompose rapidly; research showed that 20% of clipping carbon decomposed within seven days [106]. Fresh plant residues, including grass clippings and roots, make up the labile soil carbon pool. Law et al. [100] reported that after two years, plots with grass clippings returned had a 3.3% increase in labile soil carbon (826 vs. 800 mg C kg^−1^) and a 3.3% increase in total soil carbon (24.7 vs. 23.9 g C kg^−1^) relative to those with clippings collected. Additionally, returning clippings can reduce the need for fertilization [42,112], which can decrease the HCCs associated with fertilizer production and transportation. In scenarios when turfgrass clippings were removed and composted on site or elsewhere, the carbon captured in the clippings should not be considered a complete loss (Figure 1) because compost may be added to other systems, such as vegetable gardens, or used to make compost fertilizers. In some rare scenarios, such as when clippings were burnt [28], the carbon captured in the clippings was released to the atmosphere as CO_2_.

### 7.4. Plant Growth Regulator

Limited research has been conducted on plant growth regulator (PGR) effects on carbon sequestration in turfgrass. López-Bellido et al. [51] found that the application of paclobutrazol and trinexapac-ethyl (both PGRs inhibit gibberellin biosynthesis) to creeping bentgrass fairway turf increased SOC. Because paclobutrazol promotes root growth, the authors [51] also determined that the SOC concentration was higher with paclobutrazol applications in comparison with no PGR for all soil depths between 0 and 15 cm. In contrast, N applications increased SOC concentration only near the soil surface (0–2.5 cm depth) in the same study [51]. Trinexapac-ethyl had a lesser effect in promoting carbon sequestration than paclobutrazol [51].

## 8. Methods for Carbon Research and Limitations

Although knowledge of the complete carbon footprint of turfgrass systems is still limited, many studies in the literature provide useful information with respect to how turfgrass contributes to net carbon sequestration or emissions by analyzing soil samples, photosynthesis, respiration, etc. Direct measurement of all inputs and outputs of a turfgrass–soil system is challenging and sometimes not feasible. Most urban research has been conducted in residential lawns by collecting soil samples and correlating results with homeowner surveys; such a method also assumes that a turfgrass system within the residential lot is the same age as the house. Quantifying SOC in turfgrass systems over time can be useful, but seasonal SOC variation needs to be considered when determining sampling time. Unlike managing other crop systems on a monthly basis, turfgrass management practices, such as mowing and irrigation, are conducted on a weekly or even daily basis. Many turfgrass carbon studies have revealed seasonal variations in SOC, CO_2_ flux, and biomass measurements [29,40,43,50,58,113]; therefore, research needs to be conducted over a long period of time, i.e., one or more years.

Net ecosystem CO_2_ exchange can be measured on a small scale with a sealed gas chamber or on a large scale with the eddy covariance method. Quantifying NEE with a sealed clear chamber has been limited in turfgrass research [113,114]. Although many studies have measured soil respiration with sealed gas chambers [29,31,35,40,55,56,58,59,61,64,66], among those studies, only one also measured the photosynthesis rate [56]. Additionally, research continuously measuring CO_2_ fluxes in turfgrass systems is very limited. Livesley et al. [68] used automatic chambers to measure CH_4_ and N_2_O fluxes for three weeks. In a recent study, Velasco et al. [28] continuously monitored flux gradient using CO_2_ sensors over a few years. The eddy covariance method was used on larger turfgrass areas, such as urban landscape [36] and sod farm [88], but has limitations to use on small turf areas [115]. Ng et al. [55] used both eddy covariance and flux chambers to quantify carbon balance in a tropical turfgrass system.

Models are useful for simulation of medium- to long-term (100 to <1000 years) changes, which are nearly impossible to monitor in field studies. Many models have been developed to predict GHG emissions in agriculture. A few studies have estimated carbon cycling in turfgrass systems by using model simulations, such as the CENTURY model [37,42,92,111], the DAYCENT model [102], the DNDC (DeNitrification–DeComposition) biogeochemical model [10], and other life cycle analysis models [8,76]. On a nationwide scale, Milesi et al. [5] used the Biome-BGC ecosystem process model to simulate carbon balance of turfgrasses in the USA.

Tracking soil carbon changes over a long period of time is not always feasible. To better understand the long-term dynamics of SOC, Bandaranayake et al. [92] applied the CENTURY model to turfgrass systems and estimated carbon sequestration in the 0–20 cm layer at the rate of 0.9 to 1.2 Mg C ha^−1^ yr^−1^ on golf course fairways for about 30 years and 0.6 Mg C ha^−1^ yr^−1^ on putting greens for 34 to 44 years. They also showed that the CENTURY model correlated well with historic soil-testing data generated by Qian and Follett [21]. The CENTURY model is a multicompartmental ecosystem model that was developed to evaluate carbon dynamics in the Great Plains grasslands [116]. The major input variables for the CENTURY model include soil texture, monthly air temperatures, precipitation, irrigation, lignin content of the plant, C and N contents of plant tissue and initial soil, and soil N inputs through fertilization and atmospheric deposition [116,117]. However, Trammell et al. [37] suggested no relationship between initial CENTURY model simulations and observed soil carbon and demonstrated that the CENTURY model could be improved by incorporating human disturbances and management practice factors. Qian et al. [42] showed that the CENTURY model was able to estimate annual clipping yield of Kentucky bluegrass. Similar to the CENTURY model, the DAYCENT model uses a daily time scale and includes soil water and temperature dynamics [118]. The DAYCENT model has been successfully adopted in turfgrass research to investigate long-term irrigation and fertilization effects [102] and to estimate N_2_O emissions [72]. Limited research using DAYCENT and DNDC models suggests that there is a need to further develop, improve, and validate these models specifically for turfgrass systems.

Although biochemical simulation models (such as CENTURY, DAYCENT, and DNDC) are commonly used in agriculture systems, their use in turfgrass systems is scarce. Future research is needed to more accurately estimate the whole-system carbon exchange using simulation models. Most studies in turfgrass evaluate some form of soil carbon; however, research on CO_2_ fluxes and the total carbon budget in turfgrass systems is limited. Chronosequence studies evaluate the effects of age by collecting soil samples from turfgrass sites varying in age, although this method cannot exclude the initial soil properties (including SOC). The biometric approach estimates NEE by measuring the NPP of annual shoot and root growth and subtracts R_eco_; however, this method is very labor-intensive. Alternatively, many years of measurements are needed to assess SOC changes as influenced by management practices because carbon change in soil is a slow process. Therefore, studies monitoring long-term SOC dynamics are also needed.

## 9. Best Management Practices for Carbon Sequestration

The goal of enhancing carbon sequestration in turfgrass systems can be achieved by increasing carbon fixation and decreasing CO_2_ emissions. The major emissions from turfgrass systems comprise of HCCs from operations and maintenance. Additionally, turfgrass can emit CO_2_ into the atmosphere under stress conditions when respiration exceeds photosynthesis. Therefore, proper management practices are crucial to keep HCCs low but also maintain healthy turf.

Irrigation, fertilization, and mowing are primary practices that can be optimized to promote carbon sequestration. Irrigation regimes need to be developed based on the local climate to irrigate only when rainfall is insufficient to maintain healthy turf. Irrigation increases both SOC additions and decomposition. Evapotranspiration (ET)-based irrigation can be useful to avoid overwatering but still maintain turf quality for high NPP and SOC accumulation; additional research is needed to determine the range of ET replacement for different turfgrass species to enhance carbon sequestration. Nitrogen fertilization needs to be reduced as the age of the turfgrass stand increases [10,102]. One major concern associated with N fertilization is N_2_O emissions, which have a higher GWP than CO_2_. Both overwatering and fertilization can result in N_2_O emissions, which offset the carbon sequestration potential of turfgrass systems. Therefore, fertilization efficiency should not be neglected by turf managers and homeowners to avoid intensifying the greenhouse effect. Reduced irrigation and controlled-release forms of N fertilizers are recommended to reduce N_2_O emissions in turfgrass [11]. When irrigation and fertilization inputs are low, reduced mowing needs should be expected, which saves fuel without sacrificing turfgrass quality and health. On the other hand, when turfgrass is actively growing, avoiding mowing is not an appropriate management practice. Alternatively, more energy efficient mowers (battery- and electricity-powered mowers, as well as manual reel mowers) can be used in some turfgrass systems to reduce the HCC of fuel emissions. Higher mowing height within the optimal mowing height range determined according to turfgrass species, as well as returning clippings, can also contribute to enhancing carbon sequestration. Golf courses, as a whole, have the potential to act as carbon sinks; the focus should be to reduce the HCCs of turfgrass maintenance practices from diesel and gasoline.

Another critical source of carbon losses from the turfgrass system is ecosystem respiration. Research shows that the combination of high soil moisture and temperature can boost soil microbial activities to decompose SOC, which are reflected as high ecosystem respiration [28,29,40,58]. Other organic management practices incorporating carbon into turfgrass soils, such as adding biochar and compost, need to be explored. Adding compost to lawns can increase SOC, but at the same time it also increases soil respiration [119,120]. Research evaluating the effects of management practices on minimizing ecosystem respiration is lacking.

Finally, selecting appropriate turfgrass species that are well adapted to the local climate can save significant maintenance carbon costs associated with irrigation, fertilization, mowing, and pesticides. Planting turfgrass varieties that are adapted to local conditions, as well as those tolerant to environmental (cold, heat, drought, etc.) and biotic (diseases, insects, etc.) stresses can ensure healthy turf with a longer growing season and a shorter period of dormancy, resulting in increased capacity to assimilate CO_2_. Although extensively managed turfgrasses for sports fields and putting greens may not be reliable carbon sinks, other moderately or minimally managed turf areas are potential sinks of atmospheric CO_2_. Future research needs to focus on reducing HCCs associated with turfgrass management, as well as other GHGs, such as N_2_O.

## Figures and Tables

**Table 1:** Carbon sequestration rate unit conversion.

Unit	To Covert Other Units to Mg C ha^−1^ yr^−1^, Multiply by
Mg CO_2_ ha^−1^ yr^−1^	0.2727
kg CO_2_ ha^−1^ yr^−1^	0.0002727
kg C ha^−1^ yr^−1^	0.001
kg CO_2_ m^−2^ yr^−1^	2.727
kg C m^−2^ yr^−1^	10
g CO_2_ m^−2^ yr^−1^	0.002727
g C m^−2^ yr^−1^	0.01
Mg CO_2_ km^−2^ yr^−1^	0.002727

**Table 2:** Carbon sequestration in turfgrass systems compared with other systems.

Reference	Location	Comparison *
Carbon gain in the system		
Acuña E. et al. [50]	Central Chile	SOC: turfgrass > bare soil
Bae and Ryu [59]	Seoul, South Korea	SOC: mixed forest > wetland > lawn > bare soil
Upadhyay et al. [64]	Varanasi, India	SOC: urban plantation ≈ lawn> agriculture ≈ grassland > bare soil
Bowne and Johnson [66]	Elizabethtown, PA, USA	SOC: lawn ≈ corn field
Burghardt and Schneider [26]	Ruhr, Germany	SOC: vegetable garden ≈ lawn > meadow
Byrne et al. [65]	Central PA, USA	SOC: lawn ≈ bark > unmanaged vegetation> gravel
Campbell et al. [27]	Virginia, USA	Soil carbon: forest ≈ lawn
Golubiewski [34]	Colorado, USA	SOC: turfgrass ≈ tree SOC: urban green space > native grassland > agricultural field
Huyler et al. [67]	Auburn, AL, USA	SOC (only at 0–15 cm): lawn with tree > lawn without tree
Livesley et al. [68]	Victoria, Australia	SOC: wood chip mulched bed ≈ lawn
Livesley et al. [30]	Melbourne, Australia	SOC: tree > fairway
Raciti et al. [20]	Baltimore, MD, USA	SOC: lawn > forest
Singh et al. [69]	Knoxville, TN, USA	SOC: unmanaged system > lawn >row crop
Pouyat et al. [7]	Baltimore, MD, USA	SOC: lawn ≈ urban forest > rural forest
Pouyat et al. [7]	Denver, CO, USA	SOC: lawn > native grassland
Weissert et al. [29]	Auckland, New Zealand	SOC: parkland > urban forest
Kaye et al. [35]	Fort Collins, CO, USA	SOC: lawn > native grassland > corn ANPP: corn > lawn > native grassland
Jo and McPherson [12]	Chicago, IL, USA	Biomass: tress & shrubs> turfgrass > herbaceous plants
Groffman and Pouyat [70]	Baltimore, MD, USA	Atmospheric CH_4_ uptake: rural forest > urban forest > lawn
Livesley et al. [68]	Victoria, Australia	Atmospheric CH_4_ uptake: wood chip mulched bed > lawn
Kaye et al. [71]	Fort Collins, CO, USA	Atmospheric CH_4_ uptake: native grassland > lawn
van Delden et al. [9]	Samford Valley, Australia	Atmospheric CH_4_ uptake: forest > turfgrass > fallow > pasture
**Carbon loss in the system**		
Bae and Ryu [59]	Seoul, South Korea	R_s_: mixed forest > wetland ≈ lawn > bare soil
Ng et al. [55]	Singapore	R_s_: lawn > bare soil
Upadhyay et al. [64]	Varanasi, India	R_s_: lawn > grassland ≈ urban plantation > agriculture > bare soil
Bowne and Johnson [66]	Elizabethtown, PA, USA	R_s_: lawn > corn field
Byrne et al. [65]	Central PA, USA	Mean R_s_: lawn ≈ bark > unmanaged vegetation ≈ gravel
Decina et al. [60]	Boston, MA, USA	R_s_: urban landscape > lawn > urban forest
Livesley et al. [68]	Victoria, Australia	R_s_: wood chip mulched bed ≈ lawn
Kaye et al. [35]	Fort Collins, CO, USA	R_s_: lawn > corn ≈ native grassland
Weissert et al. [29]	Auckland, New Zealand	R_s_: parkland ≈ urban forest

* Systems were ranked from high to low; ≈ indicates that the former had a higher mean or median but was not statistically different from others at *p* < 0.05 level. SOC, soil organic carbon; ANPP, aboveground net primary productivity; R_s_, soil respiration.

**Table 3:** Soil organic carbon (SOC) accumulation rates reported in previous studies.

Reference	Turf Use	Location	Turf Age (Year)	Soil Depth (cm)	Regression Response	Number of Years to Reach Max SOC *	SOC Accumulation Rate (Mg C ha^−1^ yr^−1^)
Townsend-Small and Czimczik [23]	Lawn	Irvine, CA	2–33	20	Linear	33	1.4
Raciti et al. [20]	Lawn	Baltimore, MD	4–44	100	Linear	44	0.82
Smith et al. [25]	Lawn	Salt Lake City, UT	7–100	40	Linear	100	0.30
Sapkota et al. [93]	Lawn	Lubbock, TX	0–63	10	Quadratic	53.6	0.21
Huh et al. [90]	Green	Palmerston North, New Zealand	5–40	25	Linear	40	0.69
Carley et al. [91]	Green	North Carolina, USA	0–25	7.6	Hyperbolic	25	0.59
Qian and Follett [21]	Green	Colorado, USA	1.5–45	11.4	Quadratic	45	1.0
Qian and Follett [21]	Fairway	Colorado, USA	4–45	11.4	Quadratic with plateau	31	0.9
Gautam et al. [49]	Fairway	Lubbock, TX	13–93	7.5	Quadratic	46.4	0.22
Shi et al. [94]	Fairway	North Carolina, USA	2–100	15	Hyperbolic	100	0.5–6
Selhorst and Lal [19]	Fairway	Central Ohio, USA	2–97	15	Quadratic	14 (0–2.5 cm)	3.55
						30 (2.5–5 cm)	
						62 (5–10 cm)	
						81 (10–15 cm)	
Selhorst and Lal [19]	Rough	Central Ohio, USA	2–97	15	Quadratic	12 (0–2.5 cm)	2.64
						24 (2.5–5 cm)	
						68 (5–10 cm)	
						91 (10–15 cm)	

* For studies in which SOC increased linearly and hyperbolically, the max SOC was reached in the oldest reported system. Numbers in parentheses indicate soil depths.

## Data Availability

Not applicable.
